# The Relationship of Functional Status of Cortisol, Testosterone, and Parameters of Metabolic Syndrome in Male Schizophrenics

**DOI:** 10.1155/2020/9124520

**Published:** 2020-10-22

**Authors:** Yi Han, Haifeng Ji, Li Liu, Yuncheng Zhu, Xixi Jiang

**Affiliations:** ^1^Navy Characteristic Medical Center of PLA, Shanghai 200052, China; ^2^Shanghai Changning Mental Health Center (Affiliated to East China Normal University), Shanghai 200335, China; ^3^Shanghai Mental Health Center, Shanghai Jiao Tong University School of Medicine, Shanghai 200030, China

## Abstract

**Background:**

The cross-sectional study is aimed at investigating the relationship between cortisol, testosterone, and metabolic characteristics among male schizophrenics.

**Methods:**

174 patients were grouped based on their risk of metabolic syndrome (MetS) into the non-MetS, high-risk-MetS (HR-MetS), or MetS groups. Metabolic indices (body mass index (BMI), mean arterial pressure (MAP), cholesterol, triglyceride, and fasting blood glucose (FBG)) were associated with cortisol and testosterone levels using correlation analysis. Multiple linear regression analysis was used to associate the correlations between the WHO Quality of Life–BREF (WHOQOL–BREF) score and the five metabolic indices.

**Results:**

The WHOQOL–BREF score for the non-MetS group significantly differed from the scores of the HR-MetS and MetS groups. The triglyceride level was positively correlated with the cortisol level, while all five metabolic indices were negatively correlated with testosterone level. Stepwise regression analysis produced a model predicting WHOQOL–BREF scores with four variables including MAP, intelligence quotient (IQ), FBG, and age. The correlation analysis then showed that there was a weak linear correlation between the testosterone level and all five metabolic indices.

**Conclusions:**

Among the five metabolic indices, the risks of hypertension and hyperglycemia are correlated with the quality of life in male schizophrenics rather than those of obesity or hyperlipidemia.

## 1. Background

Metabolic syndrome (MetS) has a higher comorbidity with mental illness [1], which is a major reason that the life expectancy of patients with schizophrenia is much lower than that of healthy people [2]. MetS is clinically similar to Cushing's disease in many aspects that may be closely associated with adrenal steroids [3], in which the cortisol level may be positively correlated with MetS [4]. Moreover, androgen promotes the balance of positive nitrogen, which has a positive biological regulating effect on blood glucose and lipid. Testosterone level is negatively associated with MetS in older men [5]. Therefore, males are less likely to gain weight and suffer from metabolic diseases in their early adulthood [6].

A previous report of the cortisol-to-dehydroepiandrosterone sulfate (C/D) ratio might predict health levels of patients [7]. Meanwhile, Smith et al. [8] drew a different conclusion that the increased cortisol-to-testosterone (C/T) ratio is positively correlated with metabolic factors such as coronary heart disease and insulin resistance syndrome, and the ratio is negatively related with chronic stress [9]. However, Gallagher et al. [10] found significant increases of cortisol and dehydroepiandrosterone sulfate (DHEA-S) in the chronic course of schizophrenia. Only when DHEA-S is transformed into dehydroepiandrosterone by the action of sulfatase in peripheral tissues can it be further transformed into testosterone to produce biological effects [11]. About 90% of the blood circulation of DHEA-S comes from the adrenal cortical reticulum, and the serum concentration is mostly used to evaluate the situation of suspected adrenal androgen overproduction. DHEA-S can be converted to either androgens or estrogens impacted by gender differences. So, to focus on downstream testosterone may be more accurate than DHEA-S by further controlling the gender variable. As far as we know, there is no specific study on the downstream C/T ratio and MetS correlation among male patients with schizophrenia in China. We tested the relationship between C/T ratio and risk of MetS of schizophrenia, to explore the role of C/T ratio and to better assess disease risk prediction.

Additionally, compared with demography and symptomatology of schizophrenia, the treatment strategies [12] and quality of life (QOL) for patients [13] are of more urgent concern for the therapeutic alliance [14]. The investigation of the above issues must be based on patients' good treatment compliance, life satisfaction, and happiness [15]. MetS is a cluster of metabolic disorders, in which many metabolic components in the body are abnormal. It is considered a high-risk factor for diabetes, cardiovascular and cerebrovascular diseases, weight gain, and impaired glucose tolerance, all of which contribute to QOL declines in schizophrenics [1]. Metabolic diseases are more common in psychiatric patients due to disturbances of glucose and lipid metabolism related to antipsychotic drugs [16], inadequate individual nutrition education [17], and self-neglect [18]. Based on the above considerations, we aimed to investigate the relationship between the MetS and C/T ratio as the primary objective and MetS and QOL as the secondary.

## 2. Methods

### 2.1. Participants and Procedure

Our participants were recruited through the outpatient service system of the Shanghai Changning Center for Disease Control, from 01/01/2015 to 06/30/2016. The mental health services of the district cover nine jurisdictions and one town. The diagnosis of schizophrenia was ascertained according to the International Statistical Classification of Diseases and Related Health Problems, Tenth Revision (ICD-10). Inclusion criteria were as follows: male, Chinese Han ethnicity, aged 18 to 60 years, Wechsler Adult Intelligence Scale − Fourth Edition (WAIS − IV) ≥ 70, and steady long-term use (more than a year) of one antipsychotic drug. Exclusion criteria were as follows: metabolic disorder(s) on medication, alcohol or drug abuse, primary neuroendocrine diseases (thyroid axis, adrenal axis, gonad axis, pituitary tumor, etc.), severe physical disease(s) comorbidity, and having taken drugs for other disease(s) within two weeks.

**Figure 1:**
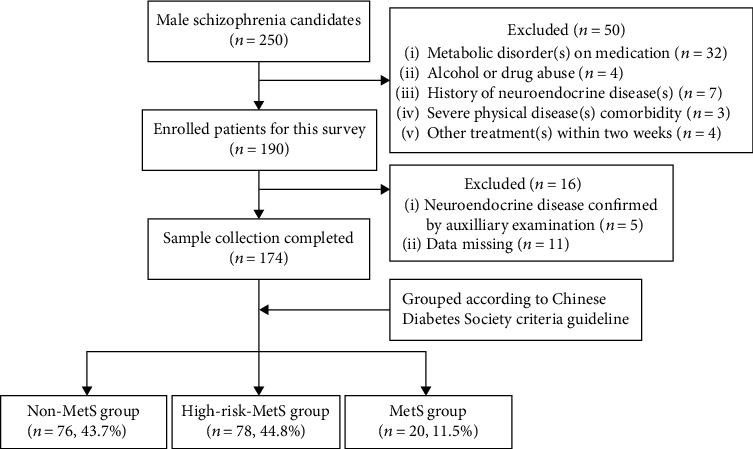
Flowchart of the screening process and data classification. We enrolled 174 male schizophrenics for each group from 250 candidates over those exclusion criteria. These cases were grouped by disease severity of MetS (76 cases in non-MetS group, 78 cases in high-risk-MetS group, with one or two of the above metabolic disorders, and 20 cases in MetS group, with three or more metabolic disorders).

The enrolled patients were divided according to the Chinese Diabetes Society criteria guideline for MetS as follows [19]: (1) overweight and/or obese: body mass index (BMI) ≥ 25.0 kg/m^2^; (2) hyperglycemia: fasting blood glucose (FBG) ≥ 6.1 mmol/l and/or 2 − hour postprandial blood glucose ≥ 7.8 mmol/l, and/or having been diagnosed with diabetes or on medication; (3) hypertension: blood pressure ≥ 140/90 mmHg and/or having been diagnosed with hypertension or on medication; and (4) dyslipidemia: fasting blood triglycerides ≥ 1.7 mmol/l and/or fasting blood high − density lipoprotein (male) < 0.9 mmol/l. The outpatient service system followed the database of patients with single medication, among which 250 patients met the requirements and agreed to participate. There were 32 cases (12.8%) on medication for at least one metabolic disorder: twenty-seven cases (10.8%) had been diagnosed as no less than one of the metabolic disorders and five cases (2.0%) as MetS (three or more metabolic disorders). We then analyzed the remaining 174 cases (70%) who met the above criteria enrolled in the study and completed the investigation (see Figure 1 for a flow diagram of sample selection).

Our study was approved on 01/01/2015 by the Institutional Ethical Committee for clinical research of Shanghai Changning Mental Health Center, Shanghai, China. Written informed consent was provided according to the *Declaration of Helsinki* and obtained from participants or their next of kin.

### 2.2. Measures

#### 2.2.1. Wechsler Adult Intelligence Scale-Fourth Edition (WAIS-IV)

The WAIS-IV can be used to assess the intelligence quotient (IQ) level for schizophrenia [20]. With an average IQ of 100, the higher the total IQ score, the higher the overall cognitive ability of the individual will be when compared with the same group of people.

#### 2.2.2. Positive and Negative Syndrome Scale (PANSS)

The Chinese Mandarin version of the PANSS [21] has been shown to be a reliable and valid instrument for the assessment of the severity of psychopathology in schizophrenics. The scale consists of 30 items, each rated using a 7-point scale. We recorded patients' total PANSS and subscales: positive symptoms, negative symptoms, and general psychopathology scores.

#### 2.2.3. Body Mass Index (BMI) and Mean Arterial Pressure (MAP)

The calculation of BMI is the weight (kg) of the individual divided by the squared height (m^2^). It is the current international standard to measure the body fat and fitness level of humans. BMI is an objective and reliable value when comparing and analyzing individuals' weight on different heights [22]. In China, the normal range is from 18.5 to 25.0 kg/m^2^. Underweight is below the normal value while overweight is above it.

MAP is the average arterial blood pressure during a cardiac cycle. The mean arterial pressure of normal adults ranged from 70 to 105 mmHg (MAP = 1/3 systolic blood pressure + 2/3 diastolic blood pressure).

#### 2.2.4. WHO Quality of Life–BREF (WHOQOL–BREF)

WHOQOL–BREF has good reliability, validity, and international comparability, which can be used for the assessment of QOL of MetS in schizophrenia [23]. The scale consists of 26 items, each rated using a 5-point scale including physiological, psychological, social, and environmental fields. The 3rd, 4th, and 26th items were adjusted by reverse scoring. Scores range from 26 to 130, with higher scores indicating better QOL.

#### 2.2.5. Haemoconcentration of Cortisol, Testosterone, FBG, Triglycerides, and Cholesterol

Nutrition consultation according to individuals' base levels was conducted to provide professional energy intake recommendations. Blood samples were then collected after patients had been conforming to the recommendations for at least one month. Fasting blood test was taken at 7 a.m. Electrochemical luminescence immunoassay was performed using Roche Modular E170 (cortisol and testosterone) [24] and Modular P800 (FBG, triglycerides, and cholesterol) [25] automatic electrochemiluminescence immunoassay system, provided by Shanghai Lanwei Clinical Testing.

#### 2.2.6. Statistical Analysis

All statistical computations were performed using SPSS 22.0. All measurement values were confirmed for normality by means of the Kolmogorov-Smirnov test and homogeneity test of variance. Normal distribution data were represented as mean (±SD). Differences in age, course of disease, IQ, WHOQOL–BREF, cortisol, testosterone, and C/T were analyzed using analysis of variance. Metabolic indices (BMI, MAP, FBG, triglyceride, and cholesterol) were associated with cortisol and testosterone levels using Pearson's correlations and hierarchical multiple linear regression analysis. Stepwise multiple linear regression analysis was carried out to examine the relationships between the WHOQOL–BREF score and the five metabolic indices, cortisol, testosterone, age, course of disease, onset age, and IQ. The probabilities for entry and removal were 0.1 and 0.15, respectively.

## 3. Results

### 3.1. Demographic Information and Clinical Features

For the 174 patients, the average age was 48.4 (±10.0) years, course of disease 23.5 (±11.4) years, and onset age 24.9 (±8.0) years. The most commonly used medications were as follows: 40 cases of olanzapine (23.0%), 29 of clozapine (16.7%), 28 of risperidone (16.1%), 15 of quetiapine (8.6%), and 14 of chlorpromazine (8.0%).

Table 1 presents the descriptive statistics for the different metabolic groups. There were no significant between-group differences in age, course of disease, onset age, IQ, and total and subscale PANSS scores (*p* > 0.05). WHOQOL–BREF scores showed significant differences between the groups (*p* < 0.01), and further post hoc LSD comparisons between the non-MetS group and the other two groups were statistically significant (*p* = 0.009/0.002), while the difference between the high-risk-MetS (HR-MetS) group and the MetS group was not (*p* = 0.153).

### 3.2. Metabolic Indices

Table 2 presents the statistics for the metabolic indices among the different patient groups. BMI, MAP, FBG, triglyceride, cholesterol, and testosterone levels and C/T ratio showed statistical differences (*p* < 0.01) between groups. There was no statistically difference in the cortisol level among the three groups (*p* = 0.110).

### 3.3. Correlations between Predictors and Metabolic Risk Factors in Male Schizophrenics

In the correlation analysis between the five metabolic indices and three predictors (cortisol, testosterone, and C/T ratio), the triglyceride level was positively correlated with the cortisol level (*r* = 0.221, *p* = 0.003), while BMI, MAP, cholesterol, and FBG levels had no correlations with cortisol level (*p* > 0.05). All five metabolic indices were negatively correlated with testosterone level (*r* [−0.343,   − 0.211], *p* < 0.05) and positively correlated with C/T ratio (*r* [0.149,  0.211], *p* < 0.05), as shown in Table 3.

To observe the interaction effect, the hierarchical multiple linear regression models of the five regression analyses were employed, shown in Table 4. The multiple linear relationships of all five metabolic indices and the two predictors were statistically significant (*p* < 0.05). The triglyceride level was positively correlated with the cortisol level (*p* < 0.05), and all five metabolic indices were negatively correlated with testosterone level (*p* < 0.05). These results were consistent with those using Pearson's correlation analysis. Furthermore, the absolute *r* values in Table 4 were higher than those of all three predictors in Table 3, especially compared to those of testosterone.

### 3.4. Predictors of WHOQOL–BREF

In this study, we attempted to associate WHOQOL–BREF scores with BMI, MAP, FBG, triglyceride, cholesterol, cortisol, testosterone, age, course of disease, onset age, and IQ through stepwise multiple linear regression. The final regression model, which comprise the predictors MAP, IQ, FBG, and age (*F*_(3,  174)_ = 4.618, *p* = 0.001, *r*^2^ = 0.099, and Δ*r*^2^ = 0.077), is shown in Table 5. The regression equation was obtained as follows: *y* = 123.705 − 0.131*x*_1_ − 0.204*x*_2_ − 1.78*x*_3_ − 0.123*x*_4_, *x*_1_ = MAP (mmHg), *x*_2_ = IQ, *x*_3_ = FBG (mmol/l), *x*_4_ = age (y).

The above regression equation showed that MAP, IQ, FBG, and age were negatively correlated with WHOQOL–BREF scores.

## 4. Discussion

Under the modern medical system, MetS is still a serious potential risk, increasing incidences of cardiovascular and cerebrovascular diseases. In the present study, we grouped schizophrenic patients according to risk of metabolic abnormalities and examined differences in age, course of disease, and IQ. We used BMI instead of waist circumference for the metabolic index as the most significant predictors of MetS for both men and women in China [26]. The study supports that any abnormality of one metabolic index can be accompanied by changes in other metabolic indices, thus aggravating the severity of disease by increasing the risk of MetS [27]. Of concern is that 32.3% of the total sample (76/235) in our district are diagnosed as MetS and 35.3% (83/235) suffer from at least one metabolic disorder. The overall time point risk rate reaches up to 67.7%. This data exceeds the overall MetS rate among male adults in China (31.0%) [28]. Schizophrenia runs a high risk of MetS comorbidity [13]. Our data also showed that the mean age of high-risk MetS in schizophrenics, 48, is younger than the 50-year-old warning line of male MetS in Shanghai [29]. The metabolic risk brought by long-term maintenance treatment must be considered in advance before determining treatment options for patients in the acute stage, rather than merely pursuing short-term treatment effects of medications [30].

The endocrine changes of adrenal and sex steroids may improve cognition in schizophrenia [31, 32]. However, testosterone secretion in males goes through a process of natural decline with the recession of testicular function. Therefore, our study sample only included male patients who were younger than 60 years old, so as to avoid male menopause with physiological “inflexion point” of testosterone [33, 34]. We excluded female patients because of physiological low levels of testosterone and hyperandrogenemia of polycystic ovarian syndrome which may interfere with the results of MetS. Under this condition, the occurrence of metabolic abnormalities is proportional to the C/T ratio and inversely proportional to the testosterone level, consistent with our expectation.

The lack of between-group difference in the cortisol level does not completely negate its predictive significance. Numerous studies have shown that elevated cortisol [35] and decreased testosterone levels [36] can be associated with MetS. Nevertheless, in our study on male patients with schizophrenia, testosterone concentration was found to have a protective effect on five metabolic indices, while cortisol concentration has a facilitation effect on an elevated triglyceride level, consistent with previous studies. For considering the potential interaction effect between the cortisol and testosterone levels, regression analyses showed that the correlation coefficients involving these two predictors were the highest among the regression models. However, the relationship between the C/T ratio and schizophrenia is complicated, relating to the course of disease, age, and duration of antipsychotic medication [31]. It is also possible that the dosage of antipsychotic drugs, which was not recorded in the present study, has an impact. For example, the commonly used low doses of quetiapine and olanzapine, as well as most atypical antipsychotic drugs, reduce cortisol levels in patients with schizophrenia [37, 38]. Obviously, these results are not satisfactory if they require patients with schizophrenia to stop taking medications, which has never happened in practice. It deserves scientific investigation because the predictiveness of the C/T ratio and its application in schizophrenia have not yet been considered in clinical assessment.

Another aim of the study is to observe whether the QOL of patients continuously decrease with increases in metabolic abnormalities. Results indicate that as long as one metabolic abnormality occurs, the QOL of patients decreases significantly, while the downtrend stops at the stage of HR-MetS and MetS. As a result, any metabolic abnormality is a risk factor for patients' QOL as a primary prevention, and it should be of high concern in tertiary prevention. In a meta-analysis, Vancampfort et al. [39] showed that the odds ratios (OR) of metabolic syndrome were induced by antipsychotics: clozapine (7.81), olanzapine (5.87), quetiapine (5.14), classical antipsychotics (4.97), risperidone (4.57), sulfenopril (3.86), and aripiprazole (3.25). Although the clinical efficacy of clozapine and olanzapine is satisfactory, the ORs of MetS caused by these two drugs are the highest. Secondly, due to insufficient family and social support and/or self-neglect to a large extent, most of the patients included in this study did not have proper medical treatment, or those metabolic disorders were even found for the first time. We should provide effective nutritional education on weight change and metabolic abnormalities, such as to encourage exercise, diet regulation, and related medical knowledge to help improve their lifestyle. For example, as a standard care, doctors could provide weight loss advice or an individual nutritional education group as a secondary prevention [17]. Finally, there was negative linear correlation between MAP and FBG levels and QOL scores. The treatment of impaired glucose tolerance and hypertension affects the patients more than obesity and hyperlipidemia. Our results support the finding that strengthening health education for self-management of diabetes and hypertension in the community could improve patients' life satisfaction [40]. Impaired glucose tolerance and hypertension have become serious factors affecting the QOL of schizophrenia [22]. Our findings support that increasing the prevention and control of these two abnormalities will achieve cost-effectiveness for tertiary preventions [41]. Stepwise multiple linear regression analysis showed that low IQ and aging are also important factors for declines in QOL. Due to their high impact across a range of domains, these two factors need to be understood and cared about by the whole society.

## 5. Conclusions

The reason may be that the effect size of schizophrenia on cortical level weighs heavily, leading to incongruency for people without psychosis [10]. Although further correlation analysis showed that cortisol level is positively correlated with triglyceride, the linearly dependent coefficient (*r*) is weak, and the predicted effect is then limited [7]. We attempted to find the C/T ratio as a prediction risk factor on MetS. Although the C/T ratio was statistically significant for the prediction, the absolute *r* values of regression model involving testosterone and cortisol levels (0.213, 0.369) were higher than those of the C/T ratio (0.149, 0.211). This suggests that the testosterone and cortisol levels may predict MetS risk factors better instead of using the C/T ratio.

In view of the high rate of MetS in patients with schizophrenia, the study of cortisol and testosterone in the assessment of patients' health status is of great significance [42]. Future studies can be conducted with expanded sample size, as well as independent studies for female subjects, so as to obtain corresponding statistical relationships between genders. Different clinical types and pharmacological effects can be used as parameters for future research.

## Figures and Tables

**Table 1:** Statistics of demographic information and clinical features of male schizophrenics across different metabolic risk groups.

	Non-MetS (*n* = 76)	HR-MetS (*n* = 78)	MetS (*n* = 20)	*F*	*p* value
Age (y)	49.6 ± 9.3	47.8 ± 10.2	46.0 ± 11.1	1.318	0.27
Course of disease (y)	24.8 ± 10.1	23.3 ± 13.0	19.6 ± 9.2	1.691	0.19
Onset age (y)	24.8 ± 7.9	24.5 ± 8.1	26.4 ± 8.3	0.452	0.64
IQ	82.6 ± 9.9	82.5 ± 8.2	84.6 ± 10.7	0.394	0.68
Total PANSS	87.3 ± 14.8	85.7 ± 11.6	83.6 ± 7.1	0.776	0.46
Positive symptoms	19.7 ± 6.3	20.0 ± 5.7	17.7 ± 4.7	1.095	0.34
Negative symptoms	18.2 ± 4.7	17.5 ± 4.9	18.6 ± 5.3	0.653	0.52
General psychopathology	38.0 ± 5.3	37.6 ± 5.4	36.5 ± 3.5	1.482	0.23
WHOQOL–BREF	82.4 ± 8.4	78.3 ± 10.0	74.9 ± 11.2	5.903	<0.01

IQ: intelligence quotient; WHOQOL–BREF: WHO Quality of Life–BREF; PANSS: Positive and Negative Syndrome Scale.

**Table 2:** Metabolic indices of male schizophrenics across different metabolic risk groups.

	Non-MetS (*n* = 76)	HR-MetS (*n* = 78)	MetS (*n* = 20)	*F*	*p* value
BMI (kg/m^2^)	21.4 ± 2.4	24.3 ± 4.1	28.2 ± 2.7	24.755	<0.01
MAP (mmHg)	87.9 ± 8.8	98.5 ± 13.5	107.5 ± 8.3	22.713	<0.01
FBG (mmol/l)	4.6 ± 0.4	5.0 ± 0.8	5.8 ± 1.0	15.776	<0.01
Triglyceride (mmol/l)	1.1 ± 0.4	1.5 ± 0.6	2.5 ± 0.4	41.657	<0.01
Cholesterol (mmol/l)	3.9 ± 0.9	4.2 ± 0.7	4.6 ± 0.8	5.129	<0.01
Cortisol (nmol/l)	450.9 ± 128.6	489.6 ± 157.7	511.9 ± 102.8	2.245	0.11
Testosterone (nmol/l)	18.8 ± 7.4	15.5 ± 7.4	12.3 ± 3.1	8.379	<0.01
C/T ratio	28.0 ± 15.0	40.5 ± 25.5	45.7 ± 19.7	9.623	<0.01

C/T ratio: cortisol-to-testosterone ratio; BMI: body mass index; MAP: mean arterial pressure; FBG: fasting blood glucose.

**Table 3:** Linear correlation analysis between predictors and metabolic risk factors in male schizophrenics.

	BMI (kg/m^2^)	MAP (mmHg)	Cholesterol (mmol/l)	Triglyceride (mmol/l)	FBG (mmol/l)
Cortisol (nmol/l) (*r*)	-0.061	0.065	0.042	0.221	0.056
*F*	0.637	0.730	0.304	8.792	0.547
*p*	0.426	0.394	0.582	0.003	0.460
Testosterone (nmol/l) (*r*)	-0.343	-0.240	-0.211	-0.327	-0.211
*F*	22.999	10.522	8.019	20.646	7.992
*p*	0.000	0.001	0.005	0.000	0.005
C/T ratio (*r*)	0.165	0.211	0.149	0.199	0.152
*F*	4.841	8.016	3.903	7.127	4.092
*p*	0.029	0.005	0.050	0.008	0.045

C/T ratio: ratio of cortisol to testosterone; BMI: body mass index; MAP: mean arterial pressure; FBG: fasting blood glucose.

**Table 4:** Hierarchical multiple linear regression analysis of cortisol and testosterone levels for each metabolic parameters.

Model	*β* ^a^	S.E.	*β* ^b^	*t*	*p*	*r*	*r* ^2^	Δ*r*^2^
BMI						-0.369	0.136	0.126
Constant	28.403	1.266		22.443	<0.001			
Cortisol	-0.003	0.002	-0.123	1.705	0.090			
Testosterone	-0.197	0.038	-0.369	5.113	<0.001			
MAP						-0.248	0.062	0.051
Constant	100.977	4.355		23.187	<0.001			
Cortisol	0.002	0.007	0.024	0.319	0.750			
Testosterone	-0.429	0.132	-0.243	3.237	0.001			
FBG						-0.214	0.046	0.035
Constant	5.238	0.264		19.874	<0.001			
Cortisol	0.000	0.000	0.021	0.277	0.782			
Testosterone	-0.022	0.008	-0.209	2.762	0.006			
Triglyceride						-0.368	0.135	0.125
Constant	1.514	0.204		7.432	<0.001			
Cortisol	0.001	0.000	0.170	2.358	0.019			
Testosterone	-0.026	0.006	-0.299	-4.142	<0.001			
Cholesterol						-0.213	0.045	0.034
Constant	4.480	0.284		15.797	<0.001			
Cortisol	0.000	0.000	0.006	0.082	0.934			
Testosterone	-0.024	0.009	-0.212	-2.798	0.006			

^a^ ^b^Unstandardized coefficients. Standardized coefficients. BMI: body mass Index; MAP: mean arterial pressure; FBG: fasting blood glucose.

**Table 5:** Results of stepwise multiple linear regression analysis for WHOQOL–BREF in male schizophrenics.

Model	*β* ^a^	S.E.	*β* ^b^	*t*	*p*	*r*	*r* ^2^	Δ*r*^2^
Constant	123.705	10.409		11.884	<0.001	-0.314	0.099	0.077
MAP	-0.131	0.045	-0.174	-2.313	0.022
IQ	-0.204	0.078	-0.194	-2.261	0.010
FBG	-1.780	0.934	-0.142	-1.906	0.058
Age	-0.123	0.072	-0.126	-1.703	0.090

^a^Unstandardized coefficients. ^b^Standardized coefficients. MAP: mean arterial pressure; FBG: fasting blood glucose.

## Data Availability

The data that support the findings of this study are available from the Shanghai Changning Mental Health Center. Restrictions apply to the availability of these data, which were used under license for this study.

## Abbreviations

MetS: Metabolic syndrome
C/T: Cortisol-to-testosterone
C/D: Cortisol-to-dehydroepiandrosterone sulfate
DHEA-S: Dehydroepiandrosterone sulfate
QOL: Quality of life
ICD-10: the International Statistical Classification of Diseases and Related Health Problems, Tenth Revision (ICD-10)
WAIS-IV: Wechsler Adult Intelligence Scale-Fourth Edition
PANSS: Positive and Negative Syndrome Scale
BMI: Body mass index
MAP: Mean arterial pressure
WHOQOL–BREF: WHO Quality of Life–BREF
FBG: Fasting blood-glucose
HR-MetS: High-risk metabolic syndrome.
